# A novel p.T139M mutation in HSPB1 highlighting the phenotypic spectrum in a family

**DOI:** 10.1002/brb3.774

**Published:** 2017-07-21

**Authors:** Jakkrit Amornvit, Mehmet E. Yalvac, Lei Chen, Zarife Sahenk

**Affiliations:** ^1^ Center for Gene Therapy The Research Institute at Nationwide Children's Hospital Columbus OH USA; ^2^ King Chulalongkorn Memorial Hospital and Department of Medicine Faculty of Medicine Chulalongkorn University Bangkok Thailand; ^3^ Department of Pediatrics and Neurology Nationwide Children's Hospital and The Ohio State University Columbus OH USA; ^4^ Department of Pathology and Laboratory Medicine Nationwide Children's Hospital Columbus OH USA

**Keywords:** α‐crystallin domain, Charcot–Marie–Tooth disease 2F clinical phenotype, congophilic aggregates, HSPB1 mutations

## Abstract

**Introduction:**

Mutations in the *HSPB1* gene encoding the small heat shock protein B1 are associated with an autosomal dominant, axonal form of Charcot–Marie–Tooth disease 2F (CMT2F) and distal hereditary motor neuropathy. Recently, distal myopathy had been described in a patient carrying HSPB1 mutation adding to the complexity of phenotypes resulting from HSPB1 mutations.

**Methods:**

Five patients in a family with concerns of hereditary neuropathy were included. Detailed clinical examinations, including assessments of motor and sensory function, and electrophysiological data were obtained. Genetic analysis was requested through a commercial laboratory. In vitro studies were carried out to assess the pathogenicity of the novel mutation found in this family studies.

**Results:**

All patients carried a novel mutation, c.146 C>T (p.T139M), substitution in the α‐crystallin domain of HSPB1 causing a clinical phenotype with hyperreflexia and intrafamilial variability, from muscle cramps as the only presenting symptom to a classic CMT phenotype. In vitro studies showed that cells expressing HSPB1‐T139M displayed decreased cell viability with increased expression of apoptosis markers. Moreover, overexpression of the mutant, not the wild‐type HSPB1, caused formation of congophilic aggregates.

**Conclusions:**

In vitro findings strongly support the pathogenicity of this novel mutation. We propose that Congo red histochemical stain may serve as a simple screening tool for investigating if the aggregates in mutant cells have misfolded β‐pleated sheet secondary structures.

## 1 INTRODUCTION

Charcot–Marie–Tooth disease (CMT) is the most common genetic neuromuscular disease, comprising a heterogeneous group of neuropathic disorders for which more than 80 causative genes have now been described (Rossor, Evans, & Reilly, 2015). Mutations in the small heat shock protein, HSPB1, have been found to cause degeneration of peripheral nerves, presenting with two major phenotypes. One is CMT type 2F (CMT2F; OMIM 606595), which is an axonal form of hereditary neuropathy characterized by progressive degeneration of motor and sensory nerves in a length‐dependent manner, causing weakness in the lower limbs affecting distal muscles, and mild to moderate sensory impairment in feet and hands. The second phenotype is distal hereditary motor neuronopathy 2B (OMIM 608634), which resembles CMT2F with progressive, symmetrical, and predominantly distal weakness, but differs having minimal or lack of sensory symptoms (Rossor, Kalmar, Greensmith, & Reilly, 2012). Recently, distal myopathy had been described in a patient carrying HSPB1 mutation (Lewis‐Smith et al., 2016) adding to the complexity of phenotypes resulting from HSPB1 mutations.

HSPB1 (also named HSP27) belongs to a group of ubiquitous molecular chaperone proteins, which are composed of a highly conserved α‐crystallin domain, a β‐sandwich structure, that is the hallmark of all proteins belonging to this family (Basha, O'Neill, & Vierling, 2012; Kappe, Boelens, & de Jong, 2010; Mymrikov, Seit‐Nebi, & Gusev, 2011). Small heat shock proteins are increased following exposure of cells to the environmental or physiological stressors. They act as molecular chaperones that counteract the formation of aberrantly folded polypeptides and allowing correct refolding during stress recovery (Mymrikov et al., 2011). HSPB1 has been thought to play an important role in maintaining neuronal cell homeostasis through stabilizing cytoskeleton, facilitating the clearance of abnormal protein accumulation through its ability to modulate the ubiquitin–proteasome pathway (Elliott et al., 2013; Parcellier et al., 2003; Treweek, Meehan, Ecroyd, & Carver, 2015). Decreased expression of HSPB1 was shown to cause impairments in growth and cytoskeletal organization (Mairesse, Horman, Mosselmans, & Galand, 1996). HSPB1 is also known to have the ability to bind and stabilize microtubules (Hino, Kurogi, Okubo, Murata‐Hori, & Hosoya, 2000; Launay, Goudeau, Kato, Vicart, & Lilienbaum, 2006); in fact, treatment with a selective HDAC6 inhibitor successfully reversed the clinical phenotype of both S135F and P182L transgenic mice (d'Ydewalle et al., 2011). In addition, HSPB1 protected cells against several proapoptotic agents when expressed at high level, while the inhibition of its expression was found to predispose cells to apoptosis (Kamada et al., 2007; Rocchi et al., 2006), presumably by triggering the release of cytochrome c (Paul et al., 2002). Similar to αB‐crystallin, HSPB1 is constitutively expressed in tissues with high rates of oxidative metabolism, including, the heart, type I and type IIa skeletal muscle fibers, brain, and spinal cord neurons (Arrigo et al., 2007; Maatkamp et al., 2004; Srivastava et al., 2012).

In the present report, we described a novel mutation, c.146 C>T (p.T139M), substitution in the α‐crystallin domain of *HSPB1* causing a clinical phenotype with hyperreflexia and intrafamilial variability and muscle cramps as the only presenting symptom. Furthermore, we carried out in vitro studies and showed that cells transfected with the mutant construct displayed decreased cell viability with increased expression of apoptosis markers and congophilic aggregate formations. These studies strongly support the pathogenicity of this novel mutation.

## 2 PATIENTS AND METHODS

### 2.1 Patients

Five patients in a family were included in this study. The proband was initially referred to the Neuromuscular Clinic at Nationwide Children's Hospital, Columbus, Ohio for an evaluation of muscle cramps. Subsequently, the other family members were directed to the clinic with concerns of hereditary neuropathy. Detail family and clinical examinations, including assessments of motor and sensory function, were obtained during outpatient visits. Muscle strength was graded according to standard Medical Research Council scale. Patients’ phenotype was defined as CMT2 based on clinical and electrophysiological data. Other relevant clinical data and investigation were also obtained from the patient records. The molecular diagnosis was first made in the index patient by requesting a diagnostic gene testing panel for autosomal dominant CMT2 through a commercial laboratory. The c.146 C>T (p.T139M) substitution in *HSPB1* was confirmed in all affected siblings subsequently.

### 2.2 In vitro studies

#### 2.2.1 Expression of wild‐type and T139M HSPB1 protein in SHSY‐5Y and HeLa cells

Plasmid vector carrying full length of wild‐type HSPB1 cDNA, driven by an elongation factor‐1 alpha promoter, was designed in our laboratory. T139M mutation was induced by using site‐directed mutagenesis kit (Agilent Technologies, USA) to obtain mutant HSPB1 vector with same backbone. All vectors were sequenced and confirmed to have right nucleotide order.

Human HeLa and SHSY‐5Y cells were grown in culture medium containing Dulbecco's modified Eagle's medium with 10% fetal calf serum and 1% penicillin and streptomycin, as a monolayer at 37°C in a humidified incubator with 5% CO_2_. SHSY‐5Y cells were transfected using electroporation, a well‐established method in our laboratory (Feng et al., 2000). Briefly, 2 × 10^6^ cells were electroporated with 10 μg of vector DNA at 200 V and 100 μF using Gene Pulser II (Bio‐Rad Laboratories, Hercules, CA, USA) were plated in six‐well plate for further analyses. HeLa cells were transfected using TransIT^®^‐LT1 (Mirus Bio LLC, Madison, WI, USA) according to the manufacturer's protocol. Transfection efficiency was determined by transfecting the cells with the same amount of control vector carrying green fluorescent protein cDNA sequence and was around 70% and 50% in SHSY‐5Y cells and in HeLa cells, respectively.

#### 2.2.2 Cell viability analysis

Cell viability after transfection was measured using CellTiter 96 MTS assay (Promega, Fitchburg, WI, USA) at 24, 48, and 72 hr after transfection. Briefly, the cells were incubated with MTS solution for half an hour, subsequently the absorbance at 490 nm was read from each well by using Biotek ELISA plate Reader (BioTek Instruments, Winooski, VT, USA). The assays were performed three times in triplicates for each group.

#### 2.2.3 RT‐PCR for apoptosis markers

Total RNA was isolated from human SHSY‐5Y cell line 24 hr after transfections using the RNeasy mini kit (Qiagen, Hilden, Germany) according to the manufacturer's protocol. qPCR experiments were performed by using iTaq^™^ universal SYBR^®^ Green supermix (Bio‐Rad Laboratories, Hercules, CA, USA). Primer sequences for apoptosis markers were designed using the Primer‐BLAST online tool (Primer‐BLAST, RRID:SCR_003095) and were as follows: TP53, forward primer 5′ ACCTATGGAAACTACTTCCTGAAA 3′, reverse primer 5′ CTGGACCTGGGTCTTCAGTG 3′; caspase‐3, forward primer 5′ GTGAGGAGTTAGCGAGCCC 3′, reverse primer 5′ TTATTAATGAGAATGGGGGAAGAGG 3′; Bax, forward primer 5′ CAGGGTGGTTGGGGGCT 3′, reverse primer 5′ GGCGTCCCAAAGTAGGAGAG 3′.

Three independent assays were done in triplicates by using ABI 7500 real‐time PCR machine and the results computed and analyzed using DataAssist^™^ Software (RRID:SCR_014969; DataAssist).

#### 2.2.4 Statistics

Data are presented by GraphPad Prism 7 (RRID:SCR_002798; Graphpad Prism) as mean values and standard error of mean bars. The number of significance is calculated by unpaired two‐tailed Student's *t*‐test with *p* values that are categorized for different significance levels: *p < .05, **p < .01, and ***p < .001.

#### 2.2.5 Transfection and in situ Congo red staining for morphological evaluation

HeLa cells were selected for these studies due to their large cytoplasmic volume. Cells transfected with mutant or wild‐type HSPB1 constructs were grown on coverslips for 24 hr and then were stained with Congo red to determine if the aggregates display amyloidogenic properties using our previously published protocol (Feng et al., 2000). Briefly, cells were first fixed with 10% formalin for 10 min and stained with 1% Congo red (Sigma‐Aldrich, St. Louis, MO, USA) for 5 min, followed by destaining with 0.01% potassium hydroxide in 50% ethanol. Coverslips were then passed through graded ethanol concentrations for dehydration and mounted in a mounting medium and examined by fluorescent microscopy under rhodamine filter.

## 3 RESULTS

### 3.1 Patients

#### 3.1.1 Clinical presentation

**Figure 1:**
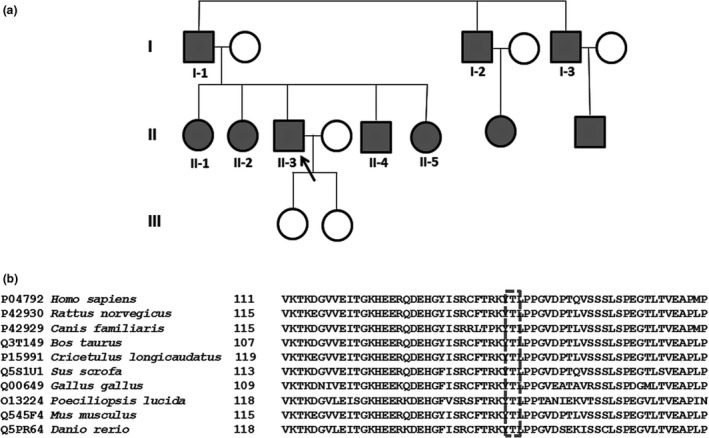
(a) Pedigree of the family. Filled square and circles indicate affected males and females, respectively. Proband II‐3 is indicated with an arrow. (b) The sequences of the HSPB1 protein from a range of divergent species, compared with multiple sequence alignment tool. The mutated region of the threonine amino acid at position 139 (highlighted with dash box) is highly conserved

Patients’ age ranged from 35 to 55 years. Progressive ankle instability and difficulty walking were the presenting symptoms in all except the proband who complained of muscle cramps. Disease onset was in the second and fourth decade in two sisters who were more severely affected with gait difficulties and foot drop. Another sister in her fifth decade had a 5‐year history of ankle instability, mild gait difficulty, and weakness in her ankle stabilizer muscles. One male sibling had disease onset late in the third decade and an 8‐year history of ankle instability and sensory symptoms The pedigree of the family is presented in Figure 1 and the relevant clinical examination findings are detailed in Table 1.

**Table 1:** Clinical findings of the affected patients carried HSPB1‐T139M

	II‐1	II‐2	II‐3	II‐4	II‐5
Age at first examination	55/F	54/F	47/M	47/M	35/F
Duration of symptoms (years)	5	>5	5	8	9
Presenting symptoms	Ankle instability	Difficulty walking, ankle instability	Muscle cramps in lower legs	Ankle instability, paresthesia in hands/feet	Difficulty walking, foot drop
Walk on heel/toe	Some difficulty	Unable	Good	Some difficulty	Unable
Hammer toes	No	No	Yes	No	Yes
Pes cavus	Cavovarus	Yes	Yes	Yes	Yes
Toe extension	Absent	Absent	Absent on the right	Absent	Absent
Toe spreading	Absent	Absent	Absent	Absent	Absent
Strength (Medical Research Council scale)					
Ankle stabilizers	4	3	5	4	2
Muscle stretch reflexes					
Biceps	Hyperreflexia	Hyperreflexia	Hyperreflexia	Hyperreflexia	Hyperreflexia
Triceps	Hyperreflexia	Hyperreflexia	Hyperreflexia	Hyperreflexia	Hyperreflexia
Brachioradialis	Hyperreflexia	Hyperreflexia	Hyperreflexia	Hyperreflexia	Hyperreflexia
Knee	Hyperreflexia	Hyperreflexia	Hyperreflexia	Hyperreflexia	Hyperreflexia
Ankle	Absent	Absent	Hyperreflexia	Absent	Absent
Pinprick sensation	↓ Below low calf	↓ Below mid‐calf	↓ Below low calf	↓ Below mid‐calf	↓ Below mid‐calf
Vibration (Rydel‐Seiffer tuning fork)					
Toe	6	5	6	Absent	6
Lateral malleolus	Normal	Normal	Normal	6.5	Normal
Index finger	Normal	Normal	Normal	Normal	Normal

F, female; M, male; ↓, decrease.

The proband II‐3, a 47‐year‐old male, was investigated initially with complaints of occasional tightness in his calves, and night cramps involving his calves and toes for approximately 5 years. In addition, he had a recent history of waking up with numbness, predominantly on the ulna side of the right upper extremity.

On examination, he had slightly elevated arches which become flat with weight bearing. The second through fourth toes showed a slight hammertoe deformity on the right. His toe extension was absent on the right side, and toe spreading was absent bilaterally. He had normal gait and stance. He was able to walk on his heels and toes with no difficulty. His manual muscle testing revealed normal strength in both distal and proximal muscle groups. Muscle stretch reflexes were brisk and symmetrical throughout without associated Hoffman, clonus, or Babinski sign. His sensory examination revealed mildly decreased pinprick appreciation below the lower calf level with gradation. Vibration was slightly diminished only at toes, 6/8 with Rydel‐Seiffer tuning fork.

The oldest sister (II‐1), age 55‐year‐old, presented with a 5‐year history of ankle instability and described a gradual loss of arches in her feet and slight varus deformity. On examination, she had prominent arches in her feet which become flat with weight bearing. A slightly asymmetric involvement of the ankle stabilizers was noted with manual muscle testing revealing grade 4 weakness of ankle dorsiflexion, eversion and inversion, worse on the right.

The second sister (II‐2), age 54‐year‐old, reported a long‐standing history (over 5 years) of progressive distal muscle wasting and weakness in lower limbs with difficulty in walking and instability. An electrodiagnostic study performed at the age of 51 had revealed the presence of significant denervation in distal leg muscles with wasting.

The proband's 47‐year‐old brother (II‐4) was symptomatic for 8 years having mild difficulty with balance and intermittent paresthesia in his hands and feet. He also experienced occasional tripping.

The youngest sister (II‐5) was 35‐year‐old and had a 9‐year history of progressive difficulty with walking and standing on heels and toes that eventually became a full foot drop. She required bilateral lightweight ankle foot orthosis (AFOs) a year before the evaluation. She denied symptoms of hand weakness.

In summary, neurological examination in this family revealed severe impairment of both anterior and posterior compartments of lower leg muscles in two siblings; two others showed mild to moderate impairment limited to dorsiflexor muscles. Spreading and extension of toes was affected in all patients. Brisk reflexes were also found in all siblings. Except for the proband, ankle reflexes were absent in all patients. Sensory loss was variable. Mild impairment of vibratory sensation (5–6 out of 8 with Rydel‐Seiffer tuning fork) was seen in the majority and all had mild small fiber sensory deficits manifesting as decreased pinprick in the distal lower leg calf with gradation in a stocking fashion. Upper extremity motor and sensory examinations were normal in all patients.

Proband's father (I‐1) and two uncles (I‐2 and I‐3) started to show gait difficulty symptoms in their 60s. Proband's father, 87‐year‐old and lived in Puerto Rico, started to have episodes of falls and subsequently was given ankle–foot orthoses. Proband's two daughters, at ages 16 and 18 were asymptomatic.

#### 3.1.2 Electrophysiological findings

Motor and sensory nerve conduction studies were carried out in four patients (II‐2, II‐3, II‐4, II‐5). In the proband II‐3, motor and sensory nerve conduction studies showed normal or borderline normal findings except bilateral reduced tibial compound motor action potential (CMAP) amplitudes. Needle electromyography revealed chronic neurogenic process with some fibrillation potentials, restricted to the gastrocnemius and tibialis anterior muscles only. Electrodiagnostic studies of II‐2, II‐4, and II‐5 showed findings compatible with motor and sensory axonal degeneration in the lower limb nerves. CMAPs of the common peroneal and tibial nerves are markedly reduced or unobtainable. Some degree of slowing found in the tibial or peroneal nerves motor conduction velocities from the studies of II‐2 and II‐4 were determined to be proportionate to the degree of axonal loss. Motor and sensory nerve conduction studies of the upper limb nerves were exclusively normal. Based on the findings of predominant peripheral axonal degeneration with normal or over 38 m/s of motor nerve conduction velocities from the upper extremity, the diagnosis of CMT type 2 was made.

#### 3.1.3 Genetic analysis

The proband II‐3 was analyzed for several CMT axonal genes as part of a panel to include *MPZ*,* NFL*,* GDAP1*,* MFN2*,* LMNA*,* RAB7*,* GARS*, and *HSPB1* sequencing. In the proband, heterozygous missense mutations were identified in *HSPB1*: c.416C>T, which predicted to substitute a threonine to a methionine at position 139 (p.T139M), and in *GARS*: c.11C>T, which predicted to substitute proline to leucine at position 4. These two identified variants in the proband were subsequently tested in other family members. Only c.416C>T variant in *HSPB1* was found in all affected family member tested (Figure 1). In addition to the proband, the c.11C>T variant in *GARS* was detected only in II‐1, and therefore considered nonpathogenic. The threonine amino acid in 139 position in *HSPB1* is highly conserved across all different species (Figure 1), and this variant was neither found in dbSNP, ExAC browser, or 1,000 genome databases. With in silico analysis by PolyPhen‐2 (Adzhubei et al., 2010), SIFT (Kumar, Henikoff, & Ng, 2009), and Mutationtaster (Schwarz, Cooper, Schuelke, & Seelow, 2014), the variant was predicted as “probable damaging” (score 0.999), “damaging” (score 100%), and “disease causing,” respectively.

### 3.2 Functional analysis of the mutant HSPB1

**Figure 2:**
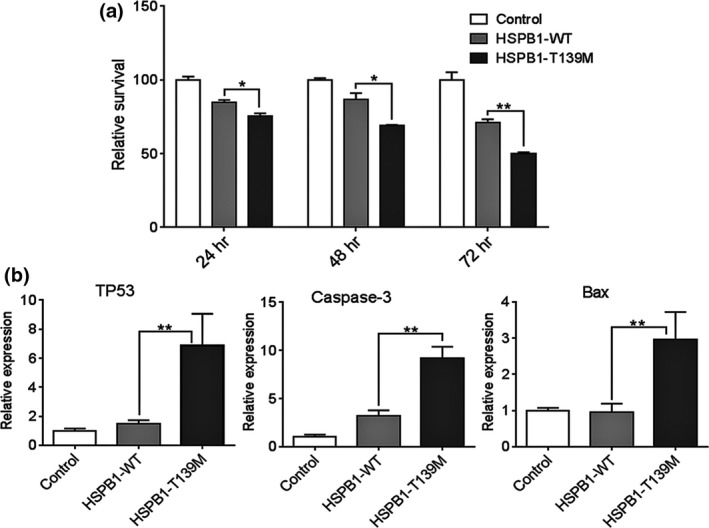
(a) SHSY‐5Y cell survival was determined 24, 48, and 72 hr after transfection with HSPB1‐WT or with HSPB1‐T139M vectors compared to the control (nontransfected cells). (b) Expression of proapoptotic genes including TP53, caspase‐3, and Bax in SHSY‐5Y cell 24 hr after transfection. Values were normalized to the control. Data shown are mean ± SEM; *p < .05, **p < .01

SH‐SY5Y is a human‐derived neuroblast‐like cell line, generally used as in vitro model for studying neuronal function and differentiation. To study the pathogenicity of HSPB1‐T139M mutation, we first evaluated the impact of this novel mutation on the overall biological fitness of this stable neuroblastoma cells using MTS cell viability assay (Cory, Owen, Barltrop, & Cory, 1991). Evgrafov et al. (2004) previously showed that N2a cells overexpressing S135F‐HSBP1 were less viable than the N2a overexpressing the wild‐type HSBP1. Similarly, we found that the expression of mutant HSBP1 lowered the cell viability significantly (Figure 2a) at all time points studied compared to HSPB1‐WT, percent survival of cells transfected with plasmid containing wild‐type HSPB1 versus mutant: 24.83 ± 2.04 versus 15.14 ± 1.46 at 24 hr (*p* < .05); 30.96 ± 0.38 versus 13.24 ± 1.67 at 48 hr (*p* < .05); and 49.77 ± 0.60 versus 28.8 ± 2.09 at 72 hr (*p* < .01).

Previous studies had shown that HSPB1 displays a neuroprotective effect through regulation of apoptosis. HSPB1 functions as antiapoptotic molecule and is implicated in various cascades of the apoptotic pathway (Acunzo, Katsogiannou, & Rocchi, 2012; Concannon, Gorman, & Samali, 2003). The major pathways mediated apoptosis regulations by HSPB1 are through inactivation of the caspase cascade and caspase‐independent apoptosis pathways via Daxx (death domain‐associated protein; Acunzo et al., 2012). In this study, we investigated the expression of the apoptotic markers to see if mutant HSPB1 decreased cell viability through apoptosis. Using real‐time PCR, we quantified the expression levels of proapoptotic genes including TP53 (tumor suppressor gene), caspase‐3 (apoptosis executor gene), and Bax (proapoptotic gene) in SH‐SY5Y cells 24 hr after transfection with HSPB1‐T139M and compared to those cells expressing the HSPB1‐WT. Results showed that the expression levels of these genes were dramatically increased in SH‐SY5Y cells transfected with the mutant suggesting that T139M substitution induced the cell death via apoptosis (Figure 2b).

**Figure 3:**
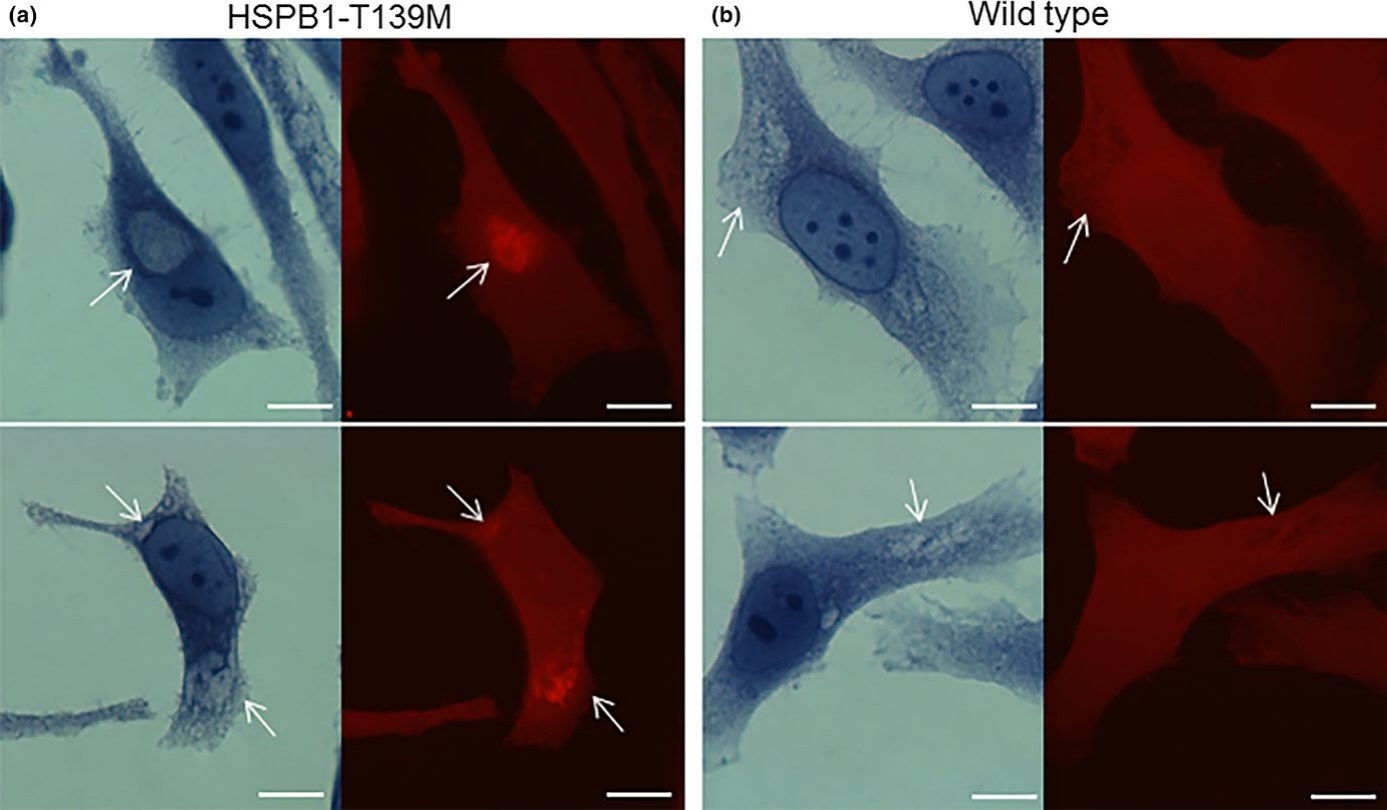
(a) HeLa cells 24 hr after transfection with HSPB1‐T139M and (b) HSPB1‐WT were stained with Congo red and examined under bright field (left side) and rhodamine optics (right side). In HSPB1‐T139M‐transfected HeLa cells, (a) displays single or multiple intracytoplasmic aggregates with congophilic properties (arrows). Bars = 10 μm

We also assessed if this novel mutation impairs protein degradation function, resulting in congophilic aggregate formations of misfolded proteins, similar to mutations in another heat shock protein, HSPB5 (αB‐crystallin) in muscle (Andley, Hamilton, Ravi, & Weihl, 2011; Selcen, 2011; Selcen & Engel, 2003). For these experiments, HeLa cells transfected with mutant or wild‐type HSPB1 constructs were grown on coverslips for 24 hr and then were stained by Congo red to determine if the aggregates display amyloidogenic properties. Cells examined under rhodamine optics revealed varying size of intracytoplasmic aggregates with congophilic properties in the HSPB1‐T139M‐expressing cells (Figure 3). Wild‐type HSPB1 overexpressing cells did not display congophilia.

## 4 DISCUSSION

We report five siblings of a family with distal, predominantly motor neuropathy due to the c.416C>T transition leading to a T139M missense mutation in *HSPB1*. Although the severity of the weakness in ankle stabilizers were highly variable, hyperreflexia was invariably present in all patients and the sensory involvement were confined to a mild decrease of pinprick appreciation and vibration. The proband in this family had a very mild phenotype, presented with occasional leg cramps and mild decrease in pinprick appreciation without detectable motor deficit, which was corroborated by the presence of normal or borderline normal motor and sensory nerve conduction studies. However, easily elicitable brisk reflexes and high‐arched feet with absent toe spreading prompted us to obtain a detailed family history and clinical examination of the available family members with neuropathy to determine if our patient represented the spectrum of a phenotype of an autosomal dominant CMT variant. Genetic analysis on the proband and the affected family members subsequently revealed a T139M mutation in *HSPB1* gene, which to our knowledge has not been reported previously. The other affected siblings all presented with a clinical phenotype compatible with axonal CMT. The disease onset varied from second to fifth decades and the disease severity correlated with the duration of symptoms although there was no correlation between clinical severity and patients’ age. In fact, the youngest patient (II‐5) had the most severe motor and sensory deficit. Motor and sensory symptoms were exclusively in the distal lower extremities.

In general, one of the cardinal features of classic CMT phenotype is hyporeflexia resulting from peripheral axon degeneration and the muscle cramps are well recognized symptom of neuropathic process. Hyperreflexia, observed in this T139M phenotype, serves as a feature for appreciating the microscopic anatomy of the disease process resulting from HSPB1 mutations. Previous reports have described patients with HSPB1 mutations in the paracrystallin domain having pyramidal signs (Solla et al., 2010; Stancanelli et al., 2015). Solla et al. (2010) reported a 27‐year‐old man from a large family possessing R127W mutation in HSPB1 presented with spastic paraplegia‐like features, where other family members had phenotypes compatible with classification and diagnostic guidelines for CMT2 and dHMN. Pyramidal signs (brisk reflexes and Hoffman sign) were present in several members of a multigenerational family with A136L mutation (Stancanelli et al., 2015). E41K mutation in the N‐terminal domain of HSPB1 was also reported to present with muscle atrophy and weakness of distal extremities as well as hyperreflexia and upward plantar responses in a 39‐year‐old woman (Capponi et al., 2011). These reports are in support of our view that the disease process should be recognized as a central peripheral distal axonopathy according to the site of anatomical involvement, a classification proposed several decades ago (Schaumburg, Wisniewski, & Spencer, 1974; Spencer & Schaumburg, 1977a,b). This is reinforced by the fact that HSPB1 is ubiquitously expressed in both central and peripheral motor neurons. Moreover, we provided evidence from a transgenic animal model with R136W mutation in HSPB1, which showed neuropathological changes consistent with distal axonal pathology not only in peripheral nerves but also in the central nervous system (Srivastava et al., 2012). Axonal pathology, predominantly found in the anterior and lateral funiculus of the lumbar and sacral cords from these mice, was consistent with a length‐dependent involvement of the corticospinal tracts (Srivastava et al., 2012). In addition, other heat shock proteins, such as chaperonin 60 (heat shock protein 60) have been reported to cause prominent upper motor neuron degeneration presenting with hereditary spastic paraplegia (Fink, 2013). Mutations in other axonal CMT‐associated genes such as mitofusin‐2 (MFN2) and alanyl‐tRNA synthetase expressed in the both central and peripheral neurons, although reported rarely were associated with pyramidal signs (Motley et al., 2015).

We found that p.T139M mutation in *HSPB1* is highly conserved across all different species and the variant was predicted as disease causing with high probability. We further investigated the pathogenicity of this mutation with in vitro studies, which showed that the expression levels of apoptotic genes were dramatically increased in SH‐SY5Y cells transfected with the mutant suggesting that T139M substitution induced the cell death via apoptosis. In addition, we have shown that overexpression of the HSPB1‐T139M mutant in HeLa cells causes formation of congophilic aggregates. Congo red histochemical dye has the ability to bind specifically to crossed β‐pleated sheet structures. Wild‐type HSPB1 should maintain protein homeostasis by binding proteins in non‐native conformations, thereby preventing substrate aggregation. The T139M mutant, however, failed in this function and resulted in an accumulation of misfolded proteins, which were targeted by Congo red for intercalating between the β‐pleated sheet structures.

In conclusion, our study showed that the novel mutation c.416C>T; p.T139M in HSPB gene is pathogenic and caused phenotypic variability among the siblings in a family. The clinical examination findings are compatible with a disease process presenting as central–peripheral distal axonal disease. Our study also shows that Congo red histochemical stain may serve as a simple tool to investigate if the aggregates in mutant cells have misfolded β‐pleated sheet secondary structures.

## CONFLICT OF INTEREST

None declared.
